# Sports Ability-Beliefs, Goal Orientation, and Exercise Adherence among Korean Golfers: A Causal/Multiple Mediation Model Using Phantom Variables

**DOI:** 10.5114/jhk/194132

**Published:** 2025-05-29

**Authors:** Eunchul Seo, Young-Vin Kim, Hyunkyun Ahn

**Affiliations:** 1 Department of Physical Education, Wonkwang University, Jeonbuk, Republic of Korea.; 2 Department of eSports, Division of Culture & Arts, Osan University, Gyeonggi-do, Republic of Korea.; 3 Department of Sport & Leisure Studies, Division of Arts & Health Care, Myongji College, Seoul, Republic of Korea.

**Keywords:** achievement goal orientation, motivational theories, recreational golfers, structural equation modeling

## Abstract

Continual exercise has become an important concept for mental as well as physical health, with sporting industries and communities interested in promoting exercise adherence. This study examined the causal relationship among sports ability-beliefs, achievement goal orientation, and exercise adherence among Korean recreational golfers. Data from 806 golfers were collected after Institutional Review Board deliberation and approval. To reflect the characteristics of frequent participation, golfers were limited to those who had been registered members of a golf practice facility for more than three months. The measurements included the sports ability-belief scale, the achievement goal orientation scale, and the exercise adherence scale, with evidence of construct validity based on confirmatory factor analysis. The research questions were verified through a measurement model and structural model validation. The results showed that the learning factor under incremental beliefs explained task orientation (β = 0.417, p < 0.001). Conversely, the gift factor under entity beliefs explained ego orientation (β = 0.169, p < 0.001). Thus, the significant relationship between learning and task orientation implied that consistent practice and effort in golf could enhance the positive meaning of golf participation and the perception of achievement in golfers. Conversely, the static explanation of ego orientation of gift suggested that individuals with strong beliefs in their innate golf abilities or the influence of their motor skills on golf performance experienced a sense of achievement by comparing themselves with others. Secondly, when the sub factors of sports ability-beliefs were controlled, both task orientation (β = 0.420, p < 0.001) and ego orientation (β = 0.159, p < 0.001) were statistically significant in terms of exercise adherence. Thirdly, in the relationship between sports ability-beliefs and exercise adherence, both task orientation (indirect effect: 0.074, p < 0.001) and ego orientation (indirect effect: 0.012, p < 0.05) had complete mediation effects. Ultimately, the study confirmed that the relevance of sports ability-beliefs and achievement goal orientation for exercise adherence in elite sports could also be applicable in the realm of recreational sports.

## Introduction

Exercise adherence is a concept described by Corbin and Lindsey (1994) as the degree to which individuals regularly exercise, with exercise becoming part of their daily lives. The concept of exercise adherence can provide insights into the forces behind an individual’s consistent engagement with exercise. By conceptualizing exercise adherence, we can identify and verify the variables that influence this adherence (Corbin and Lindsey, 1994). This is particularly useful when we consider it an outcome variable in sports motivation theory. By verifying variables that linearly explain exercise continuation, we can apply these in theoretical frameworks to support policies and education, and encourage ongoing participation (Kim and Seo, 2012; Oh et al., 2000). The present study focuses on golfers. Previous studies have shown that golfers’ adherence to exercise is based on various motives (e.g., goal achievement, challenge, competence, socialization, style, and health) (Jung et al., 2015), as well as unique characteristics inherent to sports competition (Jung, 2019). However, these variations in individual motives have been largely characterized through qualitative research, with the assumptions explaining exercise adherence presented from a phenomenological perspective, based on interview findings. Therefore, although the contextual process that motivates exercise adherence among golfers (golf adherence) has been recognized as a “phenomenon”, it has not been statistically validated. Thus, there is a need to develop causal models for generalization.

The most appropriate models that explain exercise adherence among golfers are based on motivational theories. Indeed, theories such as the self-determination theory (Amado et al., 2019; Deci and Ryan, 1985), theories of self-ability (Dweck, 1996; Spray et al., 2006), and the achievement goal theory (Sarrazin et al., 1996) were proposed long ago to explain characteristics of exercise adherence for both elite athletes and recreational participants. These theories conceptualize motivation for both performance and persistence, either independently or through the interaction of two or more variables (Dweck et al., 1995; Hughes and Zhang, 2007; Oh et al., 2000; Spray et al., 2006; Prończuk et al., 2023, 2024, 2024a). Among these there is the theory of sports ability-beliefs, which captures self-perceptions related to one’s beliefs about one’s exercise performance ability. This theory suggests that there are two types of beliefs related to exercise performance: entity beliefs and incremental beliefs, both of which act as motivators for exercise performance (Spray et al., 2006). First, entity beliefs refer to the perspective that one’s abilities are fixed and unchangeable. In this case, we view exercise talent as a determinant of exercise ability (Dweck, 1996). Conversely, incremental beliefs refer to the perspective that one’s abilities can be enhanced through learning and effort (Levy and Dweck, 1998). In the self-theory ability framework, individuals may perceive both entity and incremental beliefs as equally important, or a particular belief may be dominant. Investigating this topic, studies have reported that these beliefs influence exercise orientation (with the subfactors of task and ego orientation) or motivation, such as goal orientation, exercise adherence, and competence (Biddle et al., 2003; Dweck et al., 1995; Park and Kim, 2010; Skalski et al., 2024).

Indeed, Sarrazin et al. (1996) reported that athletes with incremental beliefs tended to be more task-oriented, judging success through improvement resulting from personal effort; in contrast, those who were more ego-oriented evaluated success based on their superior ability and accomplishments through comparison with others. In other words, sports ability-beliefs explain individual differences in goal orientation towards sports achievement. The overall trend of extant research suggests that the stronger the incremental beliefs, the more they explain motivation, effort, and performance persistence (Amado et al., 2019). However, considering that entity beliefs also have static effects on various variables (Biddle et al., 2003; Cury et al., 2002; Whitehead et al., 2004), more clarity is still needed on this topic. The most widely used scale today for measuring sports ability-beliefs is the Conceptions of the Nature of Athletic Ability Questionnaire 2 (CNAAQ2) proposed by Biddle et al. (2003). This scale reevaluated the Conceptions of the Nature of Athletic Ability Questionnaire (CNAAQ), which had been proposed by Sarrazin et al. (1996) several years earlier. The original CNAAQ consists of six factors related to beliefs about one’s athletic ability (stable, gift, general, learning, incremental, and special). The revised CNAAQ2 comprises only four factors (stable, gift, learning, and incremental). The factors capture the beliefs that one’s athletic ability can be perceived as *stable* and a *gift* as well as that one’s ability can improve through *learning* and become *incrementally* better. The stable and gift factors are considered dimensions of entity beliefs, whereas incremental and learning factors are regarded as components of incremental beliefs. Thus, the CNAAQ2 scale presupposes that sports ability-beliefs consist of both entity and incremental beliefs. It then defines these factors as four distinct dimensions (Biddle et al., 2003). In contrast, the achievement goal theory, which is presumed to be based on sports ability-beliefs as well, considers the dimension of achievement satisfaction from sports participation as the motivator (Duda and Nicholls, 1992; Galli and Gonzalez, 2015; Sarkar and Fletcher, 2014; Spray et al., 2006). This theory includes two influencing factors of exercise orientation: task orientation and ego orientation. Duda and White (1992) reported that individuals with high task orientation exerted more effort to refine their skills, whereas those with high ego orientation placed greater importance on innate talent. In other words, they tended to focus more on external factors, such as owning good equipment (Solomon and Bone, 1993). Furthermore, Papaioannou and Macdonald (1993) reported that individuals with high task orientation tended to focus on cooperation, skill mastery, and effort as their main purpose for sports participation; whereas those with high ego orientation were motivated to participate in sports based on social status elevation or external goal attainment.

These two factors of achievement goal theory are reported to either positively or negatively predict competence, exercise adherence, resilience, and stress in sports situations (Amado et al., 2019; Lintune et al., 1999; Spray et al., 2006; Whitehead et al., 2004). Studies have also suggested that higher task orientation statistically explains exercise adherence as well as exercise stress reduction (Cerin and Barnett, 2011; Jung, 2019; Klain et al., 2014). With regard to ego orientation, while some research suggests an increase in exercise stress (Jung, 2019), it has also been reported to have either a positive or a negative impact on exercise adherence (Klain et al., 2014; Roberts and Ommundsen, 1996). Therefore, according to these studies, task orientation and ego orientation have different effects (Duda, 1992). However, ego orientation does not consistently exhibit a positive relationship with sports-related variables. Hence, we need to evaluate the elements of the achievement goal theory considering various situational contexts (Cumming and Hall, 2004). As such, there is still a need to investigate the causal relationship between motivation and behavior. Given the motivational theories discussed thus far, sports ability-beliefs, achievement goal orientation, and exercise adherence are presumed to have causal relationships. Focusing specifically on Korea, we believe that Korean golfers’ motivation will show variations in terms of seeking a sense of achievement, a challenge or health improvement, as well as in forming social relationships, pursuing the style, and engaging in social interactions with others (Jung et al., 2015). Furthermore, while achievement goal theory suggests that task and ego orientation explains exercise adherence, previous studies have primarily focused on elite athletes, without controlling for the motivational aspects of golf and the characteristics of recreational sports. Our study fills this gap. Based on what has been discussed above, our aim was to investigate the causal relationships among sports ability-beliefs, achievement goal orientation, and exercise adherence among golfers in Korea. To that end, the characteristics of golfers from the perspective of recreational sports were examined, and the factors that contributed to sustained engagement in this particular sport were validated. The results provide valuable insight for golf marketing, instructors, and the industry, with implications for its advancement and development.

## Methods

### Study Participants

Recreational golfers were surveyed through purposive sampling by visiting 20 golf practice facilities in Korea. The sampling period was two months, from March to April 2024. To determine golf engagement, members registered at each practice facility for a minimum of three months were targeted. After approval from the golf practice facility authorities, a survey with golfers who provided informed consent was conducted. In total, 850 responses were collected, of which 44 were excluded due to incomplete or misleading responses. The demographic characteristics of participants are presented in Table 1. The study complied with ethical standards and safety regulations set forth by the Institutional Review Board of the World Association of Public Health and Beauty (approval code: 1-20170113 119-AB-N-01-16; approval date: 20 March 2024).

**Table 1:** Participants’ characteristics.

Demographics	Category	n	%
Gender	Male	405	50.25
	Female	401	49.75
Age	20s	240	29.78
	30s	291	36.10
	40s and over	275	34.12
Average score	Mean = 99.19, standard deviation = 19.65		
Purpose of golf participation	Health	94	11.66
	Friendship	329	40.82
	Networking	95	11.79
	Self-display	23	2.85
	Fun of golf	265	32.88
Total		806	100.00

### Measurement Tools

To measure sports ability-beliefs, the CNAAQ2 scale developed by Biddle et al. (2003) was employed, consisting of 12 items divided in four factors: learning, incremental, gift, and stable. The scale had already been adapted by Park and Kim (2010) and validated for use with Korean middle and high school as well as college athletes. Park and Kim (2010) validated this tool through a series of procedures, including item revision, athlete interviews, expert consensus, and confirmatory factor analysis using a sample of 418 individuals. Since the scale they developed was for use with athletes in general, some modifications were introduced to it to adapt it to the characteristics of golfers (e.g., “When I exercise vigorously...” was modified to “When I play golf vigorously...”). The response categories were measured on a five point Likert scale ranging from 1 (“strongly disagree”) to 5 (“strongly agree”).

### Achievement Goal Orientation

The scale proposed by Kim (2001) was used to measure achievement goal orientation. This scale comprises 13 items: 7 for task orientation and 6 for ego orientation, assumed to be sub-factors of achievement goal orientation as well as exercise orientation. Respondents rated their agreement on a Likert scale ranging from 1 (“not at all”) to 5 (“very much so”).

### Exercise Adherence

To measure exercise adherence, a scale introduced by Oh et al. (2000) was used. This scale identifies propensities, functionalities, and reinforcements as sub-factors based on the framework proposed by Corbin and Lindsey (1994), comprising 15 items across three factors. These items were tailored to fit the characteristics of golfers (e.g., “actively engage in golf even without special incentives or rewards” instead of “actively engage in exercise even without special incentives or rewards”). The responses were measured on a Likert scale ranging from 1 (“not at all”) to 3 (“very much so”), consistently with the original scale.

### Valiity and Reliability

Confirmatory factor analysis (CFA) and Cronbach’s α were used to ensure the reliability of the psychological construct measurements considered for the golfers: sports ability-beliefs, achievement goal orientation, and exercise adherence. The maximum likelihood (ML) method for the CFA was employed, and the model fit was assessed based on the chi square test (χ2), the Tucker Lewis Index (TLI > 0.900), the comparative fit index (CFI > 0.900), and the root mean square error of approximation (RMSEA < 0.080) (Hong, 2000). After examining the CFA model (Table 2), several items under the goal orientation and exercise adherence scales reflected multidimensionality, except for sports ability-beliefs (achievement goal orientation: five items; exercise adherence: three items). The problematic items were removed to ensure the construct validity of the model (Kline, 2011). Subsequently, a second round of CFA model validation was conducted, which indicated that the fit indices, standardized coefficients, and reliability of the model were all acceptable (Kline, 2011).

**Table 2:** Validity and reliability analysis.

Latent variable	Path	Measurement variable	B	β	t	Cronbach’ α	χ2(df)	TLI	CFI	RMSEA (90% CI)
Sports ability-beliefs										
Stable	→	SAB 1	0.604	0.692	18.101***		255.326***(48)	0.950	0.964	0.073 (0.064~0.082)
	→	SAB 2	0.558	0.624	16.484***					
	→	SAB 3	0.691	0.720	18.690***					
Gift	→	SAB 4	0.597	0.679	20.176***	0.814				
	→	SAB 5	0.743	0.825	26.307***					
	→	SAB 6	0.739	0.824	26.268***					
Learning	→	SAB 7	0.814	0.862	29.983***	0.906				
	→	SAB 8	0.803	0.878	30.966***					
	→	SAB 9	0.810	0.883	31.177***					
Incremental	→	SAB 10	0.741	0.836	28.587***	0.905				
	→	SAB 11	0.777	0.897	31.985***					
	→	SAB 12	0.789	0.885	31.328***					
Achievement-Goal Orientation										
Task	→	AG 5	0.547	0.688	20.758***	0.828	133.597***(19)	0.932	0.954	0.086 (0.073~0.101)
	→	AG 8	0.587	0.714	21.848***					
	→	AG 10	0.663	0.822	26.404***					
	→	AG 12	0.628	0.732	22.497***					
Ego	→	AG 3	0.566	0.678	19.924***	0.789				
	→	AG 6	0.491	0.570	16.039***					
	→	AG 9	0.641	0.725	21.763***					
	→	AG 11	0.697	0.789	24.285***					
Exercise Adherence										
Predisposing	→	EA 1	0.314	0.529	14.942***	0.805	184.826***(51)	0.936	0.951	0.057 (0.048~0.066)
	→	EA 3	0.461	0.723	22.103***					
	→	EA 4	0.453	0.713	21.672***					
	→	EA 5	0.421	0.681	20.415***					
	→	EA 6	0.457	0.678	20.303***					
	→	EA 7	0.338	0.522	14.746***					
Enabling	→	EA 9	0.408	0.632	17.674***	0.771				
	→	EA 10	0.369	0.597	16.479***					
	→	EA 11	0.437	0.675	19.346***					
Reinforcing	→	EA 12	0.369	0.579	14.879***	0.719				
	→	EA 14	0.318	0.535	13.645***					
	→	EA 15	0.418	0.662	17.154***					

TLI: Turker–Lewis index, CFI: comparative fit index, RMSEA: root mean square error of approximation, SAB: sports ability beliefs items, AG: achievement-goal orentaion items, EA: exercise adherence items, * p < 0.05, *** p < 0.001; assessed through a confirmatory factor analysis

### Data Processing

The data collected were processed using SPSS (Version 23; IBM Corp., Armonk, NY, USA) to verify normality. Structural equation modeling (SEM) was performed using Amos (Version 23; IBM Corp., Armonk, NY, USA) to assess the fit and path effects of the research model. Additionally, a significance level of α = 0.05 was set for analyses. The specific analytical procedures were as follows: first, the research model was configured with sports ability-belief as the exogenous variable, and achievement goal orientation and exercise adherence as the endogenous variables. Park and Kim (2010) set the pathways according to the theoretical background of sports ability-belief, where factors related to incremental beliefs such as learning and incremental outcomes were designated to explain task orientation, while factors related to entity beliefs such as believing one’s ability as stable and a gift were designated to explain ego orientation. The two-step approach introduced by Anderson and Gerbing (1988) was used in our validation procedure, which involved first verifying the measurement model by setting the model to a saturated status and then proceeding to validate the structural model if no issues arose during the first step. Additionally, in the estimation of the model (as in the CFA model), the ML approach was used, with model fit assessed using χ2, TLI, CFI, and RMSEA (Hong, 2000).

Second, to examine the mediating effect of achievement goal orientation on the relationship between sports ability-beliefs and exercise adherence, the bootstrapping method with bias-corrected confidence intervals (Shrout and Bolger, 2002) and the phantom model approach proposed by Macho and Ledermann (2011) were used. Our mediation model fell under the category of multi-mediation by default. Unfortunately, there was an issue with using the Amos program, as noted by Macho and Ledermann (2011), since it only allowed for the estimation of bootstrapping for the total indirect effect, but not for specific indirect effects. To address this, Macho and Ledermann (2011) proposed a method that used a phantom model in the Amos program to estimate specific indirect effects. In this approach, the estimated coefficients were set to be the same as those in the original model, allowing the total effect of the phantom model to be estimated as a specific indirect effect of the original model. Therefore, even when setting up the phantom model, the coefficients remained fixed to those of the original model, resulting in no additional estimated variables. In other words, adding the phantom model to the original model did not generate additional estimates, and thus, the χ2, df, and fit indices were calculated in the same manner.

## Results

As previously stated, the ML method was used in the SEM; therefore, we conducted an examination of normality, a fundamental assumption for ML estimation (Kline, 2011). To do this, skewness and kurtosis, which are indicators of normality, were analyzed, and the criteria set by West et al. (1995) were applied: skewness ± 2.00 and kurtosis ± 4.00. As shown in Table 3, the range for skewness was from 0.241 to 0.219, and that for kurtosis was from 0.964 to 0.469. These results met our adopted criteria for normality, indicating that the fundamental assumption for the ML estimation was valid.

**Table 3:** Normality test.

Variables	Skewness		Kurtosis	
	Value	SE	Value	SE
Stable	0.047	0.086	0.469	0.172
Gift	0.241	0.086	0.293	0.172
Learning	0.193	0.086	0.964	0.172
Incremental	0.070	0.086	0.939	0.172
Task	0.219	0.086	0.520	0.172
Ego	0.057	0.086	0.223	0.172
Predisposing (EA1)	0.149	0.086	0.208	0.172
Enabling (EA2)	0.138	0.086	0.259	0.172
Reinforcing (EA3)	0.126	0.086	0.618	0.172

SE: standard error, EA: exercise adherence assessed through descriptive statistics

### Measurement Model

Anderson and Gerbing (1988) proposed a two-step SEM approach, where step one involves validating the measurement model, and step two the structural model. In the first step, the model is set to a saturated state before validating the structural model. This allows for the examination of potential issues arising during the process and the configuration of the relationships between latent and observed variables. Following this approach, the measurement model set was initially validated according to the saturated relationships derived from the theoretical frameworks. Table 4 presents these results. The results of the measurement model validation (χ2 = 675.684; *p* < 0.001; df = 209; TLI = 0.940; CFI = 0.951; RMSEA = 0.053) indicated that the model adequately explained the data. Moreover, all standardized coefficients for the latent variables explaining the measurement variables were above 0.5, indicating the excellent explanatory power of the latent variables. Thus, the results suggested that our measurement model was highly suitable (Anderson and Gerbing, 1988; Kline, 2011), prompting validation of the structural model in the next step.

**Table 4:** Measurement model.

Latent variable	Path	Measurement variable	B	β	*t*
Stable	→	SAB 3	1.000	0.719	reference
	→	SAB 2	0.814	0.628	13.439***
	→	SAB 1	0.874	0.691	13.951***
Gift	→	SAB 6	1.000	0.824	reference
	→	SAB 5	1.004	0.824	22.573***
	→	SAB 4	0.809	0.680	19.116***
Learning	→	SAB 9	1.000	0.882	reference
	→	SAB 8	0.993	0.879	34.328***
	→	SAB 7	1.005	0.861	33.020***
Incremental	→	SAB 12	1.000	0.887	reference
	→	SAB 11	0.981	0.895	35.455***
	→	SAB 10	0.939	0.837	31.451***
Task	→	AG 5	1.000	0.694	reference
	→	AG 8	1.064	0.714	18.092***
	→	AG 10	1.173	0.802	19.974***
	→	AG 12	1.166	0.749	18.864***
Ego	→	AG 11	1.000	0.784	reference
	→	AG 9	0.919	0.721	19.057***
	→	AG 6	0.721	0.579	15.317***
	→	AG 3	0.823	0.683	18.119***
Exercise adherence	→	EA 1	1.000	0.758	reference
	→	EA 2	1.144	0.792	17.832***
	→	EA 3	0.882	0.645	15.995***
χ2 = 675.684***, df = 209, TLI = 0.940, CFI = 0.951, RMSEA = 0.053					

SAB: sports ability beliefs items, AG: achievement-goal orientation items, EA: exercise adherence sub-factor, TLI: TurkerLewis index, CFI: comparative fit index, RMSEA: root mean square error of approximation, *** p < 0.001; assessed through a structural equation model

### Structural Model

**Figure 1:**
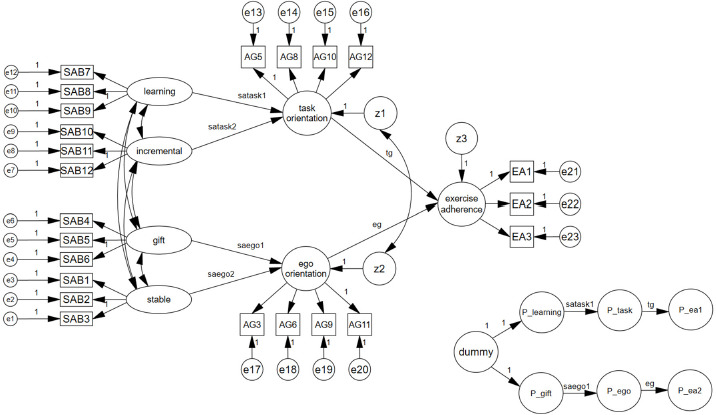
Structural model diagram applying the phantom model approach. SAB: sports ability beliefs items, AG: achievement-goal orientation items, EA: exercise adherence sub-factor

To verify the theoretical relationships among sports ability-beliefs, achievement goal orientation, and exercise adherence proposed in previous studies, a statistical model was designed, as shown in Figure 1. Specifically, the statistical model configuration entailed designating *Stable, Gift, Learning*, and *Incremental* as exogenous variables for sports ability-belief. Then, task orientation and ego orientation, considered as sub-factors of achievement goal orientation, were set as endogenous variables and mediators. Finally, exercise adherence was designated as an endogenous variable and the ultimate dependent variable.

Table 5 presents the analysis of the model presented in Figure 1, in which the adequacy of the structural model and the statistical significance of each path were assessed. First, looking at model fi, χ2 was 721.428 (*p* < 0.001), df was 217, TLI was 0.938, CFI was 0.947, and RMSEA was 0.054, indicating that the structural model was appropriate. Since the structural model adequately explained the data, the path coefficients between each variable were analyzed, and the exogenous variable (Learning) was found to be significant and influence task orientation (β = 0.417, *p* < 0.001), while Gift was also significant and affected ego orientation (β = 0.169, *p* < 0.001). However, the Incremental and Stable variables did not have statistically significant effects on task orientation and ego orientation, respectively. The sub-factors of achievement goal orientation, which were set as endogenous mediators, task orientation (β = 0.420, *p* < 0.001) and ego orientation (β = 0.159, *p* < 0.001) were statistically significant and each influenced exercise adherence. In other words, an increase in sports ability-beliefs related to the incremental belief in learning was associated with an increase in task orientation, which ultimately explained exercise adherence. Similarly, Gift (representing the entity beliefs under sports ability-beliefs) increased ego orientation and predicted exercise adherence. These findings suggest that sports ability-beliefs explain individual differences in achievement goal orientation, serving as a motivational factor for sustaining exercise. Considering the relationships among these variables, sports ability-beliefs indirectly rather than directly explain exercise adherence through the mediating effects of achievement goal orientation. As such, the statistical significance of the mediating effects (Baron and Kenny, 1986; Kline, 2011) needs to be verified.

**Table 5:** Structural equation model analysis.

Latent variable	Path	Measurement variable	B	β	*t*
Learning	→	Task	0.273	0.417	5.802***
Incremental	→	Task	0.074	0.110	1.626
Gift	→	Ego	0.156	0.169	3.616***
Stable	→	Ego	0.034	0.034	0.736
Task	→	Exercise adherence	0.269	0.420	6.832***
Ego	→	Exercise adherence	0.079	0.159	2.690**
χ2 = 721.428***, df = 217, TLI = 0.938, CFI = 0.947, RMSEA = 0.054					

** p < 0.01, *** p < 0.001; assessed through a structural equation model

To estimate the mediating effects of achievement goal orientation, the bias-corrected confidence interval bootstrapping method (Shrout and Bolger, 2002) and the phantom model approach proposed by Macho and Ledermann (2011) were used. The phantom model approach was applied to estimate specific indirect effects and their statistical significance, as shown in Table 6. The results demonstrated two specific indirect effects in the model, both of which were statistically significant. Therefore, sports ability-beliefs considering the learning and gift factors, while not directly influencing exercise adherence, positively impacted exercise adherence through the mediating effects of task and ego orientation. Thus, collectively, the two dimensions of sports ability-beliefs, namely, entity and incremental beliefs, were found to increase achievement goal orientation based on the learning and gift factors, resulting in a quantitative increase in exercise adherence.

**Table 6:** Individual indirect effect verification using the phantom model.

Mediating path	Indirect effect
Learning → task → exercise adherence	0.074***
Gift → ego → exercise adherence	0.012*

** p < 0.01, *** p < 0.001; assessed through bootstrapping (bias-corrected method)

## Discussion

The aim of the present study was to examine the causal relationships among sports ability-beliefs, achievement goal orientation, and exercise adherence in Korean golfers. Considering the distinct differences in the motivation of golfers compared with other sports, it was assumed that there would be differences with existing motivation theories. Consequently, it was hypothesized that the characteristics of this motivation would be influenced by task and ego sub-factor orientation that are part of the achievement goal orientation, which would ultimately predict exercise adherence. Specifically, the aim was to assess the characteristics of golfers from the perspective of leisure sports and derive various practical implications by explaining the characteristics that sustain golf participation. The following conclusions were derived from the results: the learning factor, corresponding to incremental sports ability-beliefs among golfers, statistically accounted for task orientation, whereas the incremental factor showed no association with task orientation.

Conversely, the gift factor, corresponding to entity sports ability-beliefs, explained ego orientation, while the stable factor did not significantly influence ego orientation. These results support prior research indicating that both entity beliefs in exercise performance ability and incremental beliefs can be motivators for exercise performance, directly explaining the direction of achievement motivation (Dweck et al., 1995; Galli and Gonzalez, 2015; Kim and Song, 2020; Sarrazin et al., 1996). Putting the present results into the context of existing theoretical concepts, the following can be asserted: first, the significant relationship between learning and task orientation implies that consistent practice and effort in golf can enhance the positive meaning of participation and the perception of achievement by golfers (Duda and Nicholls, 1992; Spray et al., 2006). Conversely, the static explanation of ego orientation by talent suggests that individuals with strong beliefs in their innate golfing abilities or the influence of their motor skills on golf performance experience a sense of achievement through comparison with others (Sarrazin et al., 1996). Several authors have pointed to the tendency to perceive competence through contact and comparison with others (Cury et al., 2002; Sarrazin et al., 1996; Solomon and Bone, 1993; Whitehead et al., 2004). Therefore, the significance of our findings lies in the verification by statistical means that the sense of competence or expectations for golf performance are associated with either consistent practice and effort or innate motor skills and talent, thereby influencing the sense of achievement associated with golf participation or the performance itself.

When connecting the findings to the motives of participants, the following was concluded: considering the demographic characteristics of participants, motivation for engaging in golf could be broadly categorized into two areas: a social role (e.g., building friendships, networking, and self-display) and a sense of competence in golf (e.g., enjoyment of golf and health management). Taking into account the participants’ characteristics and the findings of Papaioannou and Macdonald (1993), individuals with high task orientation were likely to belong to a group that perceived themselves as competent in golf, given their attitudes towards their skills and interest in the sport itself (Sarkar and Fletcher, 2014). In contrast, individuals with high ego-oriented motivation were likely to belong to a group participating in golf for socializing purposes, with motives for participation including elevating their social status or achieving external objectives (Biddle et al., 2003; Papaioannou and Macdonald, 1993). Therefore, similarly to elite athletes, participants who engaged in recreational golf also demonstrated individual differences in sports ability-beliefs and achievement goal orientation that aligned with existing theories stating that incremental beliefs are associated with task orientation and entity beliefs with ego orientation (Spray et al., 2006). Thus, considering the overall alignment with theories applied in elite sports, it can be inferred that sports ability-beliefs and achievement goal orientation are relevant variables in recreational sports as well, indicating the potential for their application in this domain.

When the sub-factors of sports ability-beliefs were controlled for, both task orientation and ego orientation significantly influenced exercise adherence. In other words, as their sense of achievement in golf increased through personal effort and improvement or through comparisons with others, motivation for exercise adherence in these individuals was strengthened. These findings suggest that, despite the differences in task orientation and ego orientation (Duda, 1992), they both serve as motivators for exercise adherence, supporting the findings of Cerin and Barnett (2011) and Klain et al. (2004). However, these results contrast with those of previous studies reporting higher likelihood of negative exercise emotions among participants with ego orientation (Lintunen et al., 1999; Whitehead et al., 2004). Most of these studies have predominantly focused on elite athletes who typically possess a significant level of experience in sports participation. In contrast with elite athletes, participants in our study were recreational golfers and therefore, did not belong to highly competitive environments. In other words, although they may have experienced a certain level of competition, this can be assumed to have been generally less intense than that experienced by elite athletes. Therefore, if they attain a certain level of achievement and competence, they are inclined to continue engaging in the sport (Corbin and Lindsey, 1994). These results suggest that the acceptance of and burden from competitive situations shape achievement motivation and perceived competence, implying that there is another motivator that influences the decision to continue or discontinue sports participation based on a certain level of acceptance (Cumming and Hall, 2004). However, as the variable of competitive situations was not controlled in our research model, future studies should incorporate competitive situations as a moderating variable.

## Conclusions

Our aim was to verify the causal relationships among sports ability-beliefs, achievement goal orientation, and exercise adherence in Korean recreational golfers. Several practical implications from the results can be identified. First, both the task orientation and ego orientation, as components of achievement goal orientation, explained exercise adherence. This was attributed to the fact that, in contrast to elite athletes, participants with characteristics typical of recreational sports do not experience excessive competition. However, the absence of a competitive environment as a variable in the model indicates the need for further research to incorporate and re-assess this aspect. Second, considering the causal relationships among sports ability-beliefs, achievement goal orientation, and exercise adherence, various approaches that consider individual differences in sports ability-beliefs are essential in golf teaching, marketing, and the golf industry to promote continuous participation (namely, exercise adherence) among golfers. For instance, individuals with entity beliefs may benefit from an emphasis on the superiority of golf as a sport to enhance their sense of achievement, whereas those with incremental beliefs may benefit from strategies emphasizing feedback and improvement in specific areas of their game to achieve the same objective. Third, it was confirmed that the relevance of sports ability-beliefs and achievement goal orientation in elite sports is also applicable in the realm of recreational sports. Specifically, incremental beliefs predicted task orientation, while entity beliefs predicted ego orientation. However, the incremental beliefs and stable factors in entity beliefs did not predict achievement goal orientation. This implies a limitation in our study, as we were unable to confirm whether this result stemmed from the characteristics of recreational sports or the nature of golf as a sport. Fourth, cultural and social factors related to the golf industry in Korea, acceptance of the sport in the country, or sustainability were not controlled for. The practice of golf has exhibited distinct patterns in Korea compared to other countries due to cultural and social circumstances (Jung et al., 2015). Therefore, in subsequent studies, it will be necessary to define cultural and social variables and validate them using statistical models that take these factors into account. Fifth, demographic and social characteristics in the causal model of sports ability-beliefs, achievement goal orientation, and exercise adherence were not considered. However, since this study aimed to explain exercise adherence among Korean golf players in general, the inclusion of a separate control group was not deemed necessary considering the original research aim. However, to devise measures that can truly benefit golf marketing, golf professionals, and the golf industry, it would be necessary to control for demographic and social characteristics. This will make possible to derive more specific and useful interpretations from the results. The empirical insights gained from this could significantly contribute to the advancement of the golf industry in Korea.
